# Methods for Quantifying Source‐Specific Air Pollution Exposure to Serve Epidemiology, Risk Assessment, and Environmental Justice

**DOI:** 10.1029/2024GH001188

**Published:** 2024-11-05

**Authors:** Xiaorong Shan, Joan A. Casey, Jenni A. Shearston, Lucas R. F. Henneman

**Affiliations:** ^1^ Department of Civil, Environmental, and Infrastructure Engineering College of Engineering and Computing George Mason University Fairfax VA USA; ^2^ Department of Environmental and Occupational Health Sciences School of Public Health University of Washington Seattle WA USA; ^3^ Department of Environmental Science, Policy, & Management School of Public Health University of California Berkeley Berkeley CA USA

## Abstract

Identifying sources of air pollution exposure is crucial for addressing their health impacts and associated inequities. Researchers have developed modeling approaches to resolve source‐specific exposure for application in exposure assessments, epidemiology, risk assessments, and environmental justice. We explore six source‐specific air pollution exposure assessment approaches: Photochemical Grid Models (PGMs), Data‐Driven Statistical Models, Dispersion Models, Reduced Complexity chemical transport Models (RCMs), Receptor Models, and Proximity Exposure Estimation Models. These models have been applied to estimate exposure from sources such as on‐road vehicles, power plants, industrial sources, and wildfires. We categorize these models based on their approaches for assessing emissions and atmospheric processes (e.g., statistical or first principles), their exposure units (direct physical measures or indirect measures/scaled indices), and their temporal and spatial scales. While most of the studies we discuss are from the United States, the methodologies and models are applicable to other countries and regions. We recommend identifying the key physical processes that determine exposure from a given source and using a model that sufficiently accounts for these processes. For instance, PGMs use first principles parameterizations of atmospheric processes and provide source impacts exposure variability in concentration units, although approaches within PGMs for source attribution introduce uncertainties relative to the base model and are difficult to evaluate. Evaluation is important but difficult—since source‐specific exposure is difficult to observe, the most direct evaluation methods involve comparisons with alternative models.

## Key Points

- Six source‐specific air pollution exposure assessment approaches are identified
- Evaluation methods are recommended for source‐specific exposure models
- Examples of exposure assessment applications in health studies are provided

## 1 Introduction

Exposure to ambient air pollution increases the risk of adverse health outcomes and mortality in humans (Brauer et al., 2019; Burnett et al., 2018; L. Henneman et al., 2023). In response to these risks, the United States has established regulations such as the National Ambient Air Quality Standards (NAAQS) (US EPA, 2014) for six criteria air pollutants: ozone (O_3_), nitrogen dioxide (NO_2_), particulate matter (<10 and 2.5 μm in aerodynamic diameter, PM_10_ and PM_2.5_), carbon monoxide (CO), sulfur dioxide (SO_2_), and lead (Pb), based on their risk‐based toxic limits. Given the health concerns and potentially high cost of regulations, a growing number of studies have sought to identify the specific sources contributing to air pollution exposure with direct applications in epidemiology (Déglin et al., 2021; Diao et al., 2021), risk assessment (Dahmardeh Behrooz et al., 2021; Thakrar et al., 2020), and environmental justice (Alvarez, 2023; Johnston & Cushing, 2020).

Exposure to air pollution from a given source is influenced by that source's emissions, physical and chemical atmospheric processes (San José et al., 2008). Additionally, social determinants of health play a role on the duration of exposure and personal intake. Researchers have employed models of varying complexity to simulate source‐specific air exposure (i.e., pollution from a specific source or source category), ranging from distance‐based metrics from individual sources (L. R. F. Henneman et al., 2019, 2021) to more complex methods that incorporate air pollution transport and chemistry (Lawal et al., 2022; Spiridonov et al., 2019). While more complex models offer sophisticated parameterizations of emissions and atmospheric processes, they typically face trade‐offs between spatial resolution and spatial coverage due to their large computational demands. These models can provide either high‐resolution local or regional modeling or coarser resolution over larger spatial scales, but not both. An additional limitation of these models is the need for expert‐level knowledge for their proper implementation and interpretation.

Source‐specific exposure metrics can either be reported in physical units (e.g., concentration) or as an indirect/scaled index (e.g., the number of nearby industrial facilities (Masroor et al., 2020) or total gasoline consumption in an exposed population density zone (Tong & Azevedo, 2020; Q. Wang et al., 2020)). Indirect/scaled indices are limited in their comparison to regulatory standards or existing concentration response functions, but they can still provide useful information for exposure assessment especially when direct measurements are not feasible due to budget, time, or data constraints.

Because source‐specific metrics are often not quantitatively evaluated against observations, the uncertainty in exposure assessed by these metrics is difficult to quantify. While ground truth observations of source‐specific exposure are possible through targeted measurement campaigns (J. Xu et al., 2022), limited spatial and temporal coverage of these observations precludes their use for evaluation population‐scale exposure models.

Techniques such as factor analysis, including positive matrix factorization (Xie et al., 2012; T. Zhang et al., 2024), are often used to derive observation‐based source‐specific exposure estimates. Unfortunately, these methods require significant interpretation by researchers and are highly sensitive to outliers that can skew results. Researchers have developed approaches for improving PMF source characterization such as dispersion‐normalization and differential concentration‐weighted trajectories (Masiol et al., 2019; Sofowote et al., 2015). Moreover, they are limited in spatial coverage, as they rely on measurements from stationary sources and do not provide the comprehensive insights that modeling‐based source apportionment approaches can offer.

We explore six source‐specific exposure model classes by their ability to (a) incorporate emissions and atmospheric processes and (b) report exposure in terms of physical units or scaled indices. In addition, we review the evaluation of these models and provide application examples in epidemiology, risk assessment, and environmental justice. The six model classes are: Photochemical Grid Models (PGMs), Data‐Driven Statistical Models, Dispersion Models, Reduced‐Complexity chemical transport Models (RCMs), Receptor Models, and Proximity Exposure Estimation Models.

Other approaches, such as satellite remote sensing (Demetillo et al., 2020; Muthukumar et al., 2022), have been increasingly used in recent years, particularly for investigating pollution sources like NO_2_. Satellite instruments such as the Tropospheric Monitoring Instrument (TROPOMI) (Goldberg et al., 2021) are used to analyze local variations in NO_2_, the Geostationary Environment Monitoring Spectrometer (GEMS) captures diurnal variation characteristics (Y. Li et al., 2023), and Tropospheric Emissions: Monitoring of Pollution (TEMPO) provides near‐real‐time air quality data (Di et al., 2020). Satellite data has been combined with chemical transport modeling to identify sources (Goldberg et al., 2017), and has been integrated into LUR (X. Yang et al., 2017) and paired with source apportionment modeling (C. J. Lee et al., 2015). Additionally, low‐cost air pollution monitoring (Idrees & Zheng, 2020) and microenvironmental modeling (Che et al., 2021) have also been employed for exposure assessments.

Existing reviews (Baker et al., 2020; Fann et al., 2012; L. R. F. Henneman et al., 2019) have described full complexity and reduced complexity approaches for assessing exposure to total air quality (e.g., all atmospheric PM_2.5_), but reviews focused specifically on source‐specific exposure approaches are lacking. Thunis et al. (2019) explored source apportionment methods, primarily focusing on quantifying incremental changes in concentrations attributable to specific sources or source categories. The authors identify three broad approaches for estimating pollution concentrations contributions from source categories at receptor locations: emissions reductions impact methods, mass transfer methods, and incremental methods.

In our analysis, emissions reductions fall under the CTM model category because of applications of sensitivity and tagging approaches that compliment emission reduction approaches. We categorize mass transfer methods and incremental methods together within a broader receptor models category because they both use information at monitors. Distinct from the overview by Thunis et al. (2019), we explore benefits, limitations, and assumptions inherit to source‐specific exposure assessment methodologies and discuss implications for epidemiology, risk assessment, and environmental justice applications.

Our study makes the following specific contributions: First, we provide a description of currently used source‐specific exposure metrics, aiding in model choice. Second, we offer insights for evaluating exposures estimated using these models. Finally, we present a framework for choosing and assessing source‐specific exposure metrics in epidemiology, risk assessment, and environmental justice.

## 2 Source‐Specific Exposure Assessment Categories

### 2.1 Source‐Specific Exposure, Epidemiology, Risk Assessments, and Environmental Justice

We explore the application of source‐specific exposure assessments in four domains: epidemiological studies, risk assessments, and environmental justice (Table 1). Air quality exposure assessments involve quantifying the degree of exposure to pollutant sources, pathways, and chemical concentrations and identifying when and how such exposure occurs (Shaddick et al., 2018; Committee on Human and Environmental Exposure Science in the 21st Century et al., 2012). A traditional exposure assessment typically involves identifying the source of interest and its emissions, as well as estimating the contributions to air pollution concentrations in the environment. Exposure at the individual level is difficult to quantify without personal monitors; therefore, ambient exposure studies often estimate population‐level aggregate concentrations.

**Table 1:** Research Domains in Which Researchers Apply Source‐Specific Air Pollution Exposure Metrics

Research domain	Goal
Exposure Assessment	Measurement or simulation of air pollutant exposure, including sources, pathways, and concentrations
Epidemiology	Analysis of the exposure [or concentration] ‐response functions between air pollutants and human diseases
Risk Assessment	Quantification of health burden associated with air pollutant exposure
Environmental Justice	Ensuring equitable air quality benefits across all community segments like race, income, or socio‐economic

Epidemiologists identify patterns, causes, and impacts of diseases in populations (Déglin et al., 2021; Shaddick et al., 2018). In air pollution epidemiology, this typically involves quantifying the association between exposure and health outcomes to establish exposure [or concentration]‐response functions (Alexeeff et al., 2015; Dionisio et al., 2016), which are often presented as relative risks or odds ratios. Source‐specific exposure metrics evaluate factors leading to health outcomes from specific pollutants or sources. Epidemiological studies frequently guide environmental policymakers in setting regulatory limits (Johns & Linn, 2011; Koning & Organization, 1987) such as the NAAQS in the US and the WHO guidelines.

Air pollution risk assessments combine information from exposure assessments, epidemiological studies and baseline health information to estimate a health burden (Déglin et al., 2021). In combination with economic valuation, risk assessments can be extended to estimate monetary burdens or benefits of specific actions. Such analysis aids in formulating preventive strategies, for example, by developing cost‐benefit analyses of proposed regulations. This practice is required for EPA regulations, even though the Clean Air Act prohibits consideration of regulatory cost (Popp, 2003). Risk assessments also inform reactive actions, such as Air Quality Alerts or fines issued by the EPA.

Environmental justice in air pollution quantifies disparities in exposure and health burdens across populations of different age, race, income, and education levels, and other factors (D’Evelyn et al., 2022; Gallagher & Holloway, 2022). These elements often converge, particularly affecting communities comprised of racially minoritized individuals, or those of lower education or lower income, communities typically situated near heavy industrial zones with significant pollution exposure (Alvarez, 2023; Rickenbacker et al., 2019).

**Figure 1:**
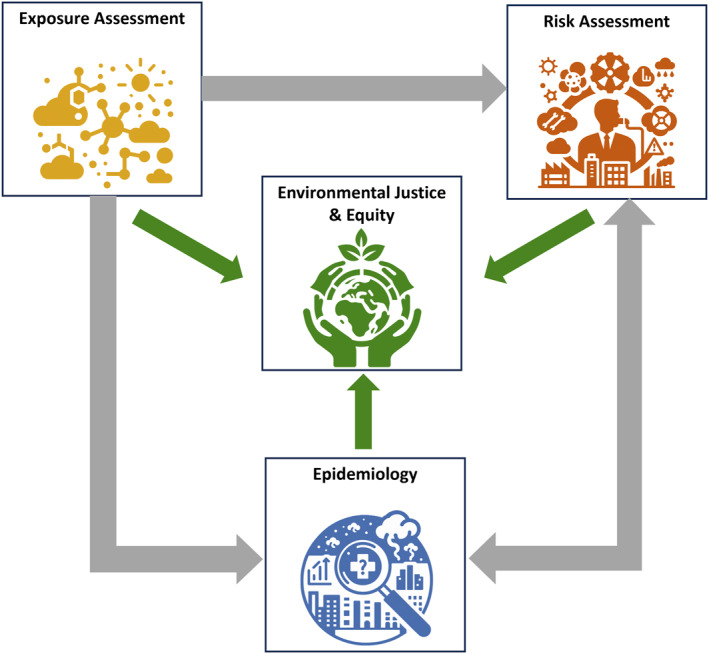
Information flow among source‐specific air pollution exposure assessment, risk assessment, epidemiology, and environmental justice research.

This concept is demonstrated in Figure 1, which outlines the interplay between exposure assessment, risk assessment, epidemiology, and environmental justice within air quality research. This figure demonstrates how data from air exposure assessments, when combined with epidemiological insights into pollution sources and risks, is used in risk assessments. Results from exposure assessments, risk assessments, and epidemiology may be used directly in environmental justice assessments (Johnston & Cushing, 2020). Researchers have argued that exposure assessments, risk assessments, epidemiological analyses, and environmental justice studies targeting individual sources yield more actionable results than quantifying impacts from air pollution from all sources (Gardner‐Frolick et al., 2022; Wambebe & Duan, 2020). Focusing on individual sources facilitates more precise policy‐making to reduce adverse health outcomes and inequities.

### 2.2 Individual‐Source Exposure Metric Design

We have identified air pollution modeling techniques that are used as source‐specific air pollution exposure metrics and have summarized (a) the approach used by each model to account for emissions and atmospheric processes, and (b) the units produced by the metrics. Our primary goal is to classify these metrics, understand the rationale for their selection, evaluate efforts made to assess their uncertainty, and make recommendations for each of the four types of studies.

### 2.3 Processes Incorporated

Three categories of processes dictate exposure to air pollution sources: emissions processes, physical processes, and chemical processes (Thakrar et al., 2020; Q. Wang et al., 2020; B. Xu et al., 2020). A fourth process category, which considers human activities such as mobility and inhalation, is important (Amato‐Lourenço et al., 2020; Faridi et al., 2021) but not considered here as we are primarily interested in interventions to reduce emissions from specific sources (source‐level interventions) rather than individual‐level interventions.

Emissions can be directly measured, as with large power plants under EPA mandates (Lavoie et al., 2017) or estimated using emission factors and activity data (Shen et al., 2021; Tainio et al., 2021). Estimated emissions generally have more uncertainty than measured emissions. Atmospheric processes that dictate pollution spread include plume rise, transport influenced by meteorology, aerosol microphysics, and wet and dry deposition (Seinfeld & Pandis, 2016). Additionally, pollutants chemically react with other chemical species, transforming into new compounds termed “secondary” air pollution species such as O_3_ and PM_2.5_.

We group models based on the approach they use to incorporate atmospheric processes into three categories: (a) first principles, (b) statistical, or (c) not explicitly considered. First principles approaches, also known as deterministic modeling, are based on a fundamental scientific understanding of the process; for example, the advection‐diffusion‐reaction equation represents a full characterization of the known processes that dictate pollutant transport and reactions in the atmosphere (Seinfeld & Pandis, 2016). While even the advection‐diffusion‐reaction equation applied in photochemical grid models involves discretization and empirical approximations, we categorize any model that is derived from first principles in this category.

First principles models include the Community Multiscale Air Quality (CMAQ), Comprehensive Air Quality Model with Extensions (CAMx) and Weather Research and Forecasting model with Chemistry (WRF‐Chems). Even with a first principles grounding, the most sophisticated models still cannot fully incorporate all known physical processes and chemical reactions.

Distinct from deterministic models, which are directly grounded in the physical understanding of atmospheric transport and chemistry, statistical models—including traditional land use regression and more flexible machine learning techniques that can better identify non‐linear relationships between inputs variables—use observed data to establish correlations between pollutant exposure and variables such as population density, land use, and proximity to emission sources (Wilkins et al., 2022; Yao et al., 2023).

### 2.4 Exposure Metric Units

We have identified two types of metrics used to quantify exposure: (a) air pollution concentration units and (b) relative non‐concentration indices (Table 2). Physical air pollution concentration units may be volumetric (e.g., parts per million by volume, ppmV) or mass‐based, such as micrograms per cubic meter (μg/m^3^). In the United States, gas concentrations are typically reported in volumetric units while particulate matter concentrations are reported in mass units (other countries tend to use mass units for gases and particles). Employing concentration units in source‐specific exposures allows for direct comparison against regulatory standards, ground‐based measurements, satellite observations, and/or outputs from other models. These comparisons should be made with an important limitation in mind: ambient air pollution observations generally do not measure source‐specific concentrations. Concentration‐response functions developed using concentration unit exposure metrics may be compared more directly to previous studies.

**Table 2:** Benefits and Limitations in the Application of Air Quality Exposure Assessments Using Physical Concentration Units or a Relative Exposure Index

Exposure assessment type	Applications	Benefits	Limitations
Physical concentration units	Compare exposure with regulatory standards and existing concentration response function	Enable development of exposure‐response function; enable exposure quantification in physical concentration units; compare exposures directly with standards	Requires air pollutant emissions as input; requires standard monitoring data for comparison; often requires models that are more time‐consuming and costly to run, with advanced technical skills needed to run the model
Relative exposure indexes	Initial hazard identification to determine the source or impact of pollutants	Enable distance comparison methods (far vs. near sources); requires reduced inputs and computational power	Inadequate for regulatory compliance; potential for additional exposure misclassification

Under a second framework, exposure assessments do not use concentration units. Instead, researchers use relative indices assumed to reflect exposure to a source. For example, researchers have used such indices to identify assumed environmental concentration variability across different geographical locations (Coudon et al., 2018), temporal spans (Zhou & Lin, 2019), or hypothetical scenarios.

An advantage of using these metrics in air pollution is their ability to translate the potentially abstract nature of air pollutant concentrations into more accessible descriptors of specific sources (e.g., number of nearby sources, or distance from a source), which may be of interest to regulators or the public. A disadvantage is the inability to compare exposure to existing standards or observed or modeled concentrations, leading to additional exposure misclassification above existing uncertainties in the model. For instance, a population living within 1 mile of a refinery may be assigned the same exposure as another population living nearby a different refinery; in contrast, a population assigned an exposure of 5 ppmV has the same exposure as a separate population being assigned 5 ppmV by the same model.

### 2.5 Spatial and Temporal Exposure Scales and Coverage

Exposure to individual sources varies in time and space. Depending on the outcome of interest and the population data available, epidemiologists (and by extension, risk assessors) can leverage both spatial and temporal variability to establish a health risk associated with a specific source or source category.

In contrast, while environmental justice studies typically rely more on spatial variability, recent advances in satellite remote sensing have allowed for the investigation of both spatial and temporal changes in air pollution related to environmental justice. For instance, (Kerr et al., 2021, 2024) used high resolution (5 × 3.5 km) TROPOMI data along with even finer (1 × 1 km) emissions and concentration data derived with satellite inputs, combined with sociodemographic data including population and race distribution, to explore how pollution distribution evolved over time in relation to vulnerable communities. Colmer et al. (2020) combined 36 years of PM_2.5_ concentrations across the U.S. with census tract and economic data to determine that the most polluted areas have remained largely unchanged over time.

As finer resolution satellite data become more available, these spatiotemporal analyses in environmental justice are likely to expand. Similar to exposure assessment methods that quantify exposure to all air pollution sources, there is often a disconnect between the temporal or spatial scale on which an exposure is modeled and the population or health data available. For example, while air quality models might provide data at an hourly resolution, health outcome data might only be available on a monthly or annual basis. Similarly, exposure models might offer high‐resolution data for a specific neighborhood, but health data might only be available at the county or city level. This disconnect has been discussed in depth elsewhere (Cui et al., 2022). In the next section, we highlight the spatial and temporal scales covered by each type of model.

## 3 Examples of Specific Models

### 3.1 Individual‐Source Exposure Model Classes

We have characterized each of the six model categories according to the their handling of the processes described above (Table 3). We categorize the models by their typical application.

**Table 3:** Source‐Specific Exposure Approaches, Categorizations, and Typical Example Applications

Model category	Emissions processes	Physical processes	Chemical processes	Exposure metric unit	Time scale	Distance scales
Photochemical Grid Models (PGMs)	First principles	First principles	First principles	Concentration	Hourly to annual	Gridded, varying from sub‐kilometer to coarser resolutions
Data‐Driven Statistical Models	Not considered	Statistical	Statistical	Concentration	Hourly to annual	Gridded, 50m+
Dispersion Models	First principles	First principles	Not considered	Concentration or relative index	Hourly to annual	Points, with potential resolution on ∼10 m scale
Reduced‐Complexity Models (RCMs)	First principles	First principles and/or statistical	First principles and/or statistical	Concentration or relative index	Daily to annual	Gridded (∼1 km) or geopolitical boundaries
Receptor Models	First principles/Statistical	Statistical	Statistical	Concentration	Hourly to daily	Monitor points
Proximity Exposure Estimation Models	First principles	Statistical	Not considered	Relative index	Long‐term	Variable

#### 3.1.1 Photochemical Grid Models (PGMs)

Photochemical Grid Models (PGMs), also referred to as chemical transport models (CTMs) or full‐complexity models, incorporate parameterizations of the key known physical‐chemical processes that determine ambient air pollution concentrations and their responses to meteorology and emissions perturbations using the advection‐diffusion‐reaction equation (Ha Chi & Kim Oanh, 2021). PGMs use an Eulerian (fixed grid) reference frame (Zheng et al., 2020). They capture the transport and transformation processes of pollutants in the atmosphere to estimate air pollution concentrations (Lawal et al., 2022; Q. Li et al., 2019). Despite uncertainty in model inputs and internal parameterizations and numerical errors, the predictive capability of these models is generally considered sufficient for regulatory applications (Emery et al., 2017), as evidenced by their ability to predict ground‐based observations for criteria air pollutants. PGMs are limited by their relatively coarse grid resolutions, though as computing power increases, finer‐resolution applications are becoming increasingly common (Visa et al., 2023).

Multiple approaches have been developed to leverage PGMs for single‐source exposure assessments. These include the brute force (or “zero‐out”) method (Kelly et al., 2015), where emissions from specific sources are set to zero to assess their impact. This method provides a direct comparison by observing how the removal of emissions from one source alters pollutant concentrations.

Adjoint approaches (Dedoussi et al., 2020; Hakami et al., 2007; Voshtani et al., 2022) estimate the marginal change in concentration across a specified region and/or period from a marginal change in emissions in each grid cell. Direct sensitivity approaches such as CMAQ's Decoupled Direct Method (CMAQ—DDM) (Baker et al., 2023) do the opposite, estimating how marginal emissions perturbations from individual source categories affect concentration levels within each model grid cell. Both approaches can simultaneously quantify the influence of numerous sources in a single model run. These approaches are particularly useful in large‐scale studies that require the assessment of multiple emission sources.

Tracer methods, such as CAMx's Ozone Source Apportionment Technology (CAMx OSAT) (Ge et al., 2021; Shu et al., 2023) and Particulate Source Apportionment Technology (PSAT) (Coelho et al., 2022; Z. Li et al., 2022), along with CMAQ's Integrated Source Apportionment Method (CMAQ—ISAM) (Kitagawa et al., 2021), track the contributions from specific sources or regions. These methods provide source attribution and identify the contribution of each tagged source category to species concentration in each model grid cell.

Evaluating adjoint, direct sensitivity, and tagging approaches is difficult because the results are generally compared against brute force results, which have similar uncertainty as the base model run along with potential additional uncertainty from changing atmospheric chemical regimes. The marginal nature of adjoint/direct sensitivity approaches suggest they are better suited for quantifying environmental justice and health outcomes changes associated with marginal emissions changes, whereas source apportionment is more suitable assessing the full burden of a given source category. However, direct sensitivity and source apportionment approaches have both been used for population‐scale exposure and environmental justice research.

PGMs are useful for source‐specific exposure assessments when coarse exposure detail is sufficient, substantial computing power is available, detailed emissions data are accessible, and there is a preference for understanding the total impacts from a source category rather than the impacts from a single source (adjoint methods can resolve impacts of all sources in a certain grid cell). Given their complexity, PGMs require substantial training to operate.

#### 3.1.2 Data‐Driven Statistical Models

Data‐driven statistical models, including Land Use Regression (LUR) and Machine Learning (ML), utilize techniques to predict pollutant concentrations based on variables such as traffic, land use, and meteorology.

LUR describes statistical models that use predictor variables to estimate pollutant concentrations at locations where direct measurements are unavailable (Doris et al., 2024). Typically, LUR incorporate meteorological data and land use factors such as weighted traffic volume, population density, physical geography (e.g., altitude) and land coverage (Hoek et al., 2008). Traditionally, land use was the main input for LUR models; however, more recent applications include a wider range of inputs, such as satellite data, CTMs outputs, and other remote sensing data to improve their accuracy. A strength of LURs is the ability of trained models to predict features at finer spatial resolutions with relatively low computational expense compared to other modeling approaches. LUR models have been applied at city, regional, and global scales (Di et al., 2019, 2020; Requia et al., 2020; van Donkelaar et al., 2015), and the resulting exposure fields have enabled large‐scale exposure assessments, epidemiological studies, and risk assessments (Burnett et al., 2018).

LUR can be applied to predict source‐specific concentrations from receptor models and chemical transport models at locations and times when such models are not available. They have been applied to estimate traffic‐related air pollution (TRAP) (Brokamp et al., 2017) and pollutants from industrial sites, restaurants (Saha et al., 2022; Q. Zhang et al., 2011), and gas production activities (Doris et al., 2024). However, LURs are not well‐suited for source‐specific analyses in scenarios where users attempt to modify independent variables to explore hypothetical “what if” situations—such as removing a road from the input data and rerunning the model. Spatial correlations among input variables can introduce confounding effects during the model training process, leading to statistically significant but potentially non‐causal relationships.

LUR models are appropriate for applications in need of high‐resolution spatial data on pollutant concentrations (but the relevant high‐resolution inputs are needed to develop such estimates). The models can provide useful information about spatial variability not available from central monitors or coarse PGM output. LURs generally do not incorporate physically based parameterizations of atmospheric weather processes, and, similar to other source‐specific models, are difficult to evaluate without observations of source‐specific exposure.

ML models extend traditional LUR by employing more flexible algorithms to uncover non‐linear relationships between predictors and source‐specific impacts. These algorithms are typically used to predict pollution levels based on variables such as weather, proximity to pollution sources, and human activity. Additionally, ML models can be used to test the sensitivity of different parameters that influence air pollution levels. Common methods for sensitivity analysis in ML include feature importance, where techniques like decision trees, random forests, or gradient boosting rank the relative importance of variables in predicting pollution levels. In an example application (Xiao et al., 2018) used an ML model to ambient PM_2.5_ concentrations using satellite‐observed Aerosol Optical Depth (AOD) with 0.25° latitude × 0.3125° longitude. While ML approaches have been used to create total PM_2.5_ exposure fields, their application in source‐specific work is still limited. We are not aware of any ML applications for predicting source‐specific exposure, and this presents a potential area for future research in source‐specific exposure studies.

#### 3.1.3 Dispersion and Trajectory Models

Dispersion and trajectory models simulate how pollutants move from their sources through the surrounding environment (Leelőssy et al., 2014). In their most basic implementation, dispersion models account for atmospheric transport of emissions from a single source. More sophisticated implementations parameterize other factors such as chemical reactions and atmospheric conditions like chemical transformations, radioactive decay, and wet deposition. Incorporation of each additional process into dispersion models has the potential to increase accuracy along with computational demand.

Dispersion models were originally designed to estimate concentrations of pollutants from individual pollutant sources, so their application is typically source‐specific. Two examples of dispersion models are the American Meteorological Society/Environmental Protection Agency Regulatory Model (AERMOD), which is used for short‐distance transport (under 10 km), and the Hybrid Single‐Particle Lagrangian Integrated Trajectory Model (HYSPLIT) (Ma et al., 2020; F. Wang et al., 2010), which is better suited for analyzing long‐range transport. Dispersion models are most often used to develop exposure estimates in concentration units, but recent application have characterized exposure using air parcel counts (L. R. Henneman et al., 2019) or as exposed/unexposed based on whether the model predicts a location is in the path of an emissions plume (Kim et al., 2020). Another example, the High‐Resolution Rapid Refresh (HRRR) Smoke model tracks smoke plumes generated by wildfires, providing forecasts of smoke impact for affected communities at different spatial resolution scales (Chow et al., 2022).

Dispersion models are useful when emissions and meteorological data are available, and they are suitable for both proximate sources using dispersion models and distant sources with trajectory models. They are most useful for quantifying concentrations of non‐reactive pollutants, like black carbon. They can generally be applied to a small number of sources with limited computing capabilities, but interpreting the inputs and results requires domain knowledge.

#### 3.1.4 Reduced Complexity Models

While all models represent a simplification of the real world, there has been increasing interest in a specific class of models broadly referred to as “Reduced Complexity Models (RCMs).” Adjoint‐based RMCs encode physical and chemical parameters within their calculated sensitivities (Henze et al., 2007; Kuylenstierna et al., 2020), enabling the models to efficiently compute how variations in input parameters, such as emissions or meteorological conditions, influence output variables, such as pollutant concentrations.

RCMs differ in how they approximate these processes, but most are trained on an initial run or series of runs of a full‐scale PGM. The models enable users to relatively quickly quantify spatial fields of total and source‐specific exposure.

The Intervention Model for Air Pollution (InMAP), Estimating Air Pollution Social Impacts Using Regression (EASIUR), and the Air Pollution Emission Experiments and Policy (APEEP, formerly AP2) models have been applied to conducting health risk and policy analysis (Baker et al., 2020). These RCMs models incorporate emissions information from all known sources—including elevated point sources and ground‐based sources like automobiles and agriculture. Other models, including the HYSPLIT Average Dispersion (HyADS) model (L. R. F. Henneman et al., 2019), estimate exposure to point sources only.

Such methods prove particularly beneficial under specific conditions: when detailed emissions data are available, when there is a need to understand exposure from specific source categories or individual sources, when only a partial representation of physical and chemical processes suffices, and when computational resources are limited. The models often assume fixed background chemistry and meteorological conditions to the period used for the initial PGM runs on which the RCMs were trained.

#### 3.1.5 Receptor Models

Receptor modeling, or source apportionment, uses observations of pollutants at a specific site to attribute these pollutants to different source categories that emit these pollutants together (Hopke, 2016). For example, wildfires emit high amounts of both potassium (K) and calcium (Ca), and co‐variation of these species in observed PM_2.5_ samples may signal influence of wildfire smoke.

In their most basic form, receptor models incorporate only information about emission source profiles (e.g., which chemicals are emitted from a given source) and the observed pollutant concentrations, although recent applications incorporate information about pollutant atmospheric transport using atmospheric dispersion. Researchers may use backward trajectory models such as HYSPLIT to evaluate their results (Chen et al., 2022). The two most popular categories of these models include factor‐based approaches such as Positive Matrix Factorization (PMF) (Gao et al., 2018; Sharma et al., 2016), and Chemical Mass Balance (CMB) (S. Lee et al., 2008).

In PMF applications, emissions source profiles (i.e., the amount of each species emitted from specific sources or source categories) are resolved using factor analysis that requires positivity in the factors. In CMB, source profiles are specified by the user rely on a pre‐established library of emission profiles. This method directly compares the measured concentrations of pollutants with the known profiles from different sources, making it a deterministic approach that depends on accurate prior knowledge of source characteristics.

Receptor models provide useful observation‐based context for source‐specific exposure. However, receptor models are sensitive to noisy measurements, outliers, and researcher interpretation. Both models have limitations in resolving large numbers of sources (they can typically resolve between 3 and 11 source categories) or pinpointing specific source locations, such as a single factory (Cesari et al., 2016).

Receptor models prove most suitable when there are high‐quality observations of many air pollution species and specific source profiles are available. Their application requires domain expertise, and it is useful to have knowledge of major air pollution emissions sources in any region that receptor models are applied to aid in interpreting model output.

#### 3.1.6 Proximity‐Based Exposure Models

Proximity‐based methodologies implicitly incorporate both stated and unstated assumptions about the relationship between pollution exposure and distance to sources (Gonzalez et al., 2022). Although these metrics do not directly capture the full complexity of emissions and atmospheric physical and chemical processes, they are generally easier to implement and often simpler to interpret.

Prevalent examples include assigning exposure based on distance to pollution sources (Aggarwal & Toshniwal, 2019; Wendt Hess et al., 2019), utilizing buffers around sources or receptors, and determining exposure by counting the number of sources within a certain distance or specified geopolitical areas (Casey et al., 2018). Refinement of these metrics for more specific air source exposure assessments is possible by integrating activity data, such as the number of vehicle miles traveled on certain roadways (Tong & Azevedo, 2020) or the operational status of a factory (L. R. F. Henneman et al., 2021). Proximity metrics can be categorized as either discrete, involving counts of nearby sources or binary exposed/unexposed classifications, or continuous, measuring the distance to the nearest source or applying inverse‐distance weighting (IDW) to account for all sources. In a representative study, Similarly, (Willis et al., 2024) examined the impact of electronic tolling on traffic dynamics and infant health outcomes in Texas. By comparing births within 500 m of tolled roads to those 2–5 km away, they suggested that reduced local traffic and air pollution from tolling led to better health outcomes.

Proximity‐based models are suitable when atmospheric modeling expertise is lacking and there is a need for readily interpretable exposure assessments. These models are also relevant for policy interventions, such as determining safe setback distances from point sources of pollution when siting facilities or building new housing, schools, or other sensitive receptors. Typically, these approaches do not require large computational resources but are limited by their inherent assumptions about exposure being directly related to proximity.

### 3.2 Evaluating Source‐Specific Exposure Metrics

There exist a few options for directly evaluating source‐specific exposure metrics. First, they can be compared against source‐specific exposure metrics from other models. This has the benefit of direct comparisons but can be limited by low availability of comparable estimates from alternative models. For example, researchers have compared CMAQ‐DDM sensitivities to brute force CMAQ (Cohan et al., 2005; Napelenok et al., 2006), and dispersion‐based and distance‐based approaches have been compared against GEOS‐Chem adjoint model results to assess individual‐source exposures (L. R. F. Henneman et al., 2021). Source impacts from a receptor model offer an additional benefit of being grounded in observations but can be limited receptor models' ability to resolve impacts from source of interest. Unfortunately, all comparisons are reliant on uncertainties in the assumed “ground truth,” meaning results should be interpreted in light of each method's overall uncertainties.

Source‐specific metrics can be evaluated indirectly through comparison with ambient observations, for example, at stationary air pollution monitors or using satellite products. These have the benefit of reflecting actual ambient conditions, but they are primarily limited in this application because they observe total concentrations, and not the portion attributable to the source of interest. To make the comparison more direct, researchers can perform evaluations with observations at locations or times that are expected to be influenced by the source of interest. In addition, researchers can upweight any evaluations made with tracer species associated with emissions only from sources of interest. For example, potassium (K) is often used as a marker for biomass burning.

Emery et al. (2017) recommend specific metrics for evaluating PGMs that can be extended to any evaluations between exposure metrics using consistent units. These include metrics assessing the magnitude of the estimated exposure: mean bias (MB), where a positive MB indicates model overestimation and a negative MB indicates underestimation; mean error (ME), where lower ME values indicate more accurate model predictions; and root mean square error, which is similar to ME, but extreme differences are weighted more heavily. MB and ME can be normalized by the mean of the “true” value and multiplied by 100% to calculate normalized mean bias (NMB) and normalized mean error, respectively, which present the error in terms of percent of the true value. An additionally informative metric is the correlation coefficient (R), which indicates the linear relationship between the variables being compared. It is recommended to calculate all of these evaluation metrics for a full understanding of uncertainty in modeled values, and quantitative evaluations should be interpreted qualitatively. Researchers have considered evaluating variability in space (Cohan et al., 2005; Napelenok et al., 2006), time (Emery et al., 2017), or space and time (L. R. F. Henneman et al., 2021).

### 3.3 Case Studies in Which Source‐Specific Exposure Assessment Models Are Evaluated

We provide two case studies to highlight evaluations of models assessing exposure to major point sources. In the first, Baker et al. (2023) justify comparisons between modeled source exposure from full‐complexity PGMs and total NO_2_ because the source of interest is a large fractional contributor to total NO_2_ in the region. In the second, L. R. F. Henneman et al. (2021), evaluate a dispersion‐based RCM model and a distance‐based metric against comparable exposure metrics from a more complex model.

#### 3.3.1 *Case Study*: Modeling Power Plant Source Impacts Using Complex Models

Baker et al. (2023) applied multiple PGMs with sensitivity tools to quantify a group of power plants' exposure contributions to NO_2_ and O_3_ in nearby communities (Baker et al., 2023). The model results—both total modeled concentrations and source‐specific contributions—were compared with ground‐based and aircraft total NO_2_ column measurements matched in time and space with the model output. Since the industrial facility was the largest source in the region, the authors justified the modeled source‐specific NO_2_ with satellite observations of total NO_2_.

In primarily qualitative comparisons of concentration maps, the authors identified which PGM sensitivity tool and grid resolution produced best matched total NO_2_ column observations. The results indicate that the modeling system's ability to depict plume behavior accurately is largely due to consistent meteorological conditions, such as wind patterns. However, the model shows limitations in capturing the plume from a facility on Lake Michigan's furthest downwind extent, likely due to the meteorological model's difficulties in accurately simulating complex wind patterns at the land‐lake interface. This issue impacts the model's capacity to fully represent dispersion processes in the afternoon, which are influenced by increased photochemical reactions.

Overall, this article shows use of varied data sources (multiple models, ground‐based observations, and aircraft observations) and evaluation approaches (e.g., qualitative comparisons of maps and assessment of maximum concentrations) needed to assess modeled source‐specific contributions.

#### 3.3.2 *Case Study*: Modeling Power Plant Source Impacts Using Reduced Complexity Models

L. R. F. Henneman et al. (2021) applied a dispersion‐based RCM and a proximity‐based exposure model to quantify exposure to each coal power plants across the United States. The authors used a PGM—GEOS‐Chem, a more sophisticated model with first principles parameterizations of emissions and physical and chemical atmospheric processes—to evaluate the simpler exposure models. GEOS‐Chem was run with the adjoint module to estimate the contribution of emissions from each grid cell to PM_2.5_ concentrations in each US state. The adjoint modeling enabled direct comparisons between facility‐specific impacts modeled by the simpler methods and the PGM.

Using quantitative evaluation metrics, they found that the RCM and proximity‐based approaches estimated similar exposure fields on an annual basis, and that they two approaches performed similarly when compared against GEOS‐Chem adjoint sensitivities. However, the performance of the proximity‐based method degraded further away from the sources relative to the RCM. This study provides a useful case study in comparing relatively simple exposure estimate approaches with a more complex first‐principles‐based model.

## 4 Conclusions

We described six model frameworks utilized for source‐specific exposure assessment. We provided suggestions for their evaluation and examples of how they can be applied in exposure, epidemiology, and risk assessment research. While most of the examples provided focus on applications in the United States, these frameworks can also be adapted for use in other regions. Most generally, decisions on which exposure assessment approach to apply depends on the source data available and importance of explicitly accounting for emissions and atmospheric processes; the need (or not) for concentration units; and the spatial and temporal scales of interest. Additional considerations include the availability of atmospheric modeling expertise and computational capacity available.

New source‐specific exposure assessment methods are being developed. In particular, satellites such as TEMPO, which is geostationary over the United States and provides fine‐scale, hourly observations, and Multi‐Angle Implementation of Atmospheric Correction (MAIAC) (Martins et al., 2017), which offers speciated observations, will provide new approaches to characterize source‐specific exposure. Since the models observe total column concentrations, results will need to be combined with other methods such as inverse emissions modeling chemical transport modeling, dispersion modeling, and receptor methods—to derive source‐specific exposure information. Inverse emissions models, for example, work by estimating the emissions responsible for observed concentrations by “inverting” the transport process and comparing observed satellite data to model predictions (Elguindi et al., 2020).

Outside the United States, the Geostationary Environment Monitoring Spectrometer (GEMS) (Q. Yang et al., 2023), a satellite‐based instrument designed to monitor air quality over East Asia in near real‐time operates from a geostationary orbit and captures high‐resolution data, such as AOD and nitrogen dioxide (NO_2_) levels, with a spatial resolution of 7 km × 8 km and hourly temporal resolution. Sentinel‐4, scheduled for launch aboard the Meteosat Third Generation Sounder (MTG‐S) satellite (Abdon et al., 2021), will also operate from a geostationary orbit over Europe. While these satellites do not directly assess source‐specific exposure, their high‐resolution data can be integrated with models like receptor or chemical transport models to enable source attribution and refine estimates of exposure linked to specific pollution sources.

We recommend that the decision to apply any source‐specific exposure metrics should be made with a priori statements about the extent that emissions, physical atmospheric processes, and chemical atmospheric processes are important for the exposure of interest, and users should establish whether it is important to assess exposure in physical units. This includes identifying the key physical processes that determine exposure and then using some of these processes to calculate an exposure metric. Researchers should acknowledge the potential information gained or lost by selecting one method over others. Evaluation is important but difficult—the most direct evaluation methods involve comparisons with alternative models, which require resources to develop.

## Conflict of Interest

The authors declare no conflicts of interest relevant to this study.

## Data Availability

Data were not used, nor created for this research.
